# Overweight and obesity at age 19 after pre-natal famine exposure

**DOI:** 10.1038/s41366-021-00824-3

**Published:** 2021-05-10

**Authors:** L. H. Lumey, Peter Ekamper, Govert Bijwaard, Gabriella Conti, Frans van Poppel

**Affiliations:** 1 Department of Epidemiology, Mailman School of Public Health, Columbia University, New York, NY USA; 2 Molecular Epidemiology Section, Biomedical Data Sciences Department, Leiden University Medical Center, Leiden, Netherlands; 3 Netherlands Interdisciplinary Demographic Institute (NIDI-KNAW)/University of Groningen, The Hague/Groningen, Netherlands; 4 Department of Economics and Social Research Institute, University College London, London, UK; 5 Institute for Fiscal Studies, London, UK

**Keywords:** Risk factors, Obesity

## Abstract

**Background:**

Weight for height has been used in the past as an indicator of obesity to report that prenatal exposure to the Dutch famine of 1944–1945 determined subsequent obesity. Further evaluation is needed as unresolved questions remain about the possible impact of social class differences in fertility decline during the famine and because being overweight is now defined by a Body Mass Index (BMI: kg/m^2^) from 25 to <30 and obesity by a BMI of 30 or more.

**Methods:**

We studied heights and weights of 371,100 men in the Netherlands born between 1943 and 1947 and examined for military service at age 19. This group includes men with and without prenatal exposure to the Dutch famine.

**Results:**

There was a 1.3-fold increase in the risk of being overweight or obese in young adults at age 19 after prenatal famine exposure in early gestation. The increase was only seen in sons of manual workers born in the large cities of Western Netherlands and not among those born in smaller cities or rural areas in the West. Social class differentials in fertility decline during the famine did not bias study results.

**Conclusions:**

The long-term adverse impact of prenatal famine on later life type 2 diabetes and mortality through age 63 is already showing at age 19 in this population as a significant increase in overweight risk.

## Key messages

- We examined the relation between undernutrition in early life and young adult body size in men born at the time of the Dutch famine of 1944–1945.
- To avoid possible biases arising from the decline in conceptions during the famine we concentrated on births to women who were already pregnant during the famine.
- We found a 1.3-fold increase in being overweight at age 19 for men exposed in early gestation but not for men exposed later in gestation. This points to an especially sensitive period in fetal development. The increase was limited to sons of manual workers, consistent with more limited access to food in this group.
- Our findings suggest that a body size increase in young adulthood foreshadows the long-term increase in later type 2 diabetes and mortality after early gestation famine.

## Introduction

Studies of the Ukraine famine of 1932–1933 [1], the Dutch famine of 1944–45 [2, 3], and three famines in 20th century Austria [4] show a relation between type 2 diabetes in later life with prenatal undernutrition. Studies of the Chinese famine of 1959–1962 have so far been less conclusive, most likely because of limitations in study design and analysis [5, 6]. Military induction records in the Netherlands have related prenatal exposure to the Dutch famine of 1944–1945 to increases in the weight for height ratio at age 19 [7]. Further examination of the military induction records could help clarify the relation between prenatal famine exposure, body size in young adults, and increased diabetes risk at a later age. This is also important as prenatal famine studies were not included in the recent International Journal of Obesity review of the effects of prenatal stress on offspring obesity [8].

The Dutch famine (Hunger Winter) of 1944–45 represents a period of civilian starvation under German occupation at the end of World War II. The food crisis developed as a culmination of conflicting Dutch, Allied and German interests [9] and provides an opportunity to study the relation between maternal nutrition in pregnancy and offspring health in a ‘quasi-experimental’ setting [10–13]. The severity and widespread nature of the famine have been fully documented [13–15]. Contemporary reports show that the famine was concentrated in the large cities of the Western Netherlands. It was limited to the last few months of the war in the period between November, 1944 and the surrender of the German forces to the Allies in May, 1945 [14, 16, 17]. After Liberation, food supplies were rapidly distributed across the country.

In a 1976 study of prenatal exposure to the Dutch famine of 1944–45, weight for height measures were used to report higher obesity rates in young adult men after prenatal famine exposure in early gestation and lower obesity rates after famine exposure in late gestation. The differences were seen in sons of manual and of non-manual workers [7].

There is a need to re-examine these findings for several reasons. First, there was a 50% decline in births conceived during the famine [13]. The decline was larger among manual compared to non-manual workers and changed the social class composition of the birth cohorts. The decline was related to food rations and was more related to social class than to the mother’s age or parity [18]. The potential confounding effect of this fertility change on reported study outcomes has never been fully examined. Second, obesity outcomes have never been examined using Body Mass Index (BMI: kg/m^2^) to define recruits as being overweight (BMI from 25 kg/m^2^ to <30 kg/m^2^) or obese (BMI of 30 kg/m^2^ or over). Third, births from smaller urban and rural conglomerations in the Western Netherlands were not examined separately, and fourth, the robustness of study results across different measures of famine exposure and different subsets of control populations needs to be established.

## Methods

### Study population

We evaluated height, weight, and BMI at age 19 in the male conscripts born between January 1, 1944 and December 31, 1947 and examined for military service in the Netherlands [7]. Physical examinations included all Dutch male citizens aged 19 years in the national population registers, except those living in psychiatric hospitals or in special institutions for the blind or for the deaf-mute (0.6%). We excluded from analysis 6.5% of men because of incomplete information on height, weight, or place of birth and 2.5% who had been born outside the Netherlands. A total of 371,100 men remain for analysis.

### Grouping by place of birth

Based on the monthly vital statistics bulletins (Maandberichten Gezondheidsstatistiek) of the Netherlands Central Bureau of Statistics from the 1930s onwards we defined as cities all municipalities listed as having a population of 25,000 or over. In 1940, 45 cities were identified nationwide [19]. Births in smaller municipalities were defined as rural. We combined this information with the region of birth to define three exposure groups: (1) Births in the ten largest Western Netherlands famine cities; (2) Births in Western Netherlands in other cities or in the rural West; (3) Other Netherlands cities and rural areas. The last group provides place controls without famine exposure (Appendix 1).

Ravelli et al. (1976) [7] had only classified as famine births the men born in the seven largest Western Netherlands famine cities, thereby ignoring births in three municipalities that were either part of urban area of The Hague or Rotterdam. They had classified as control births all men born in the areas defined as urban or urbanized rural in 1947 by the Dutch census typology of municipalities by urbanization level [20], thereby including remaining cities in the West as controls and ignoring all non-urbanized rural births [7]. Overweight and obesity study findings using the two different classification systems and study populations were then compared (Appendix 1).

### Grouping by month of birth

We grouped individuals by month of birth between January 1944 and December 1947 and defined famine exposure in relation to specific gestation periods. This was possible as gestations were shortened by no more than 3–4 days at the height of the famine [13].

The extreme famine period started in Nov 1944. We classified births between November 1944 and January 1945 as exposed in months 7–9 of gestation (Trim 3); births between February and April 1945 in months 4–6 of gestation and later (Trim 2+); and births between May and July 1945 in months 1–3 and later of gestation (Trim 1+). As the extreme famine period lasted <9 months, births exposed in the first trimester or later were not exposed throughout gestation, although the total duration of prenatal famine exposure was the longest in this group. We also classified men who were exposed to famine in early infancy (between ages 0–6 months) and found no differences with unexposed cohorts.

Further details on our grouping and on the month of birth grouping used in the Ravelli et al. obesity study [7] are provided in Appendix 2.

We compared study findings with alternative definitions of famine exposure either (a) incorporating information on distributed food rations [10], or (b) using alternative groupings by month of birth from a prior obesity study in the cohorts [7] (Appendix 2; Supplementary Table 1 and Supplementary Fig. 1).

### Socio-economic and demographic characteristics

Father’s occupation was classified as non-manual or manual as in previous studies of the cohort [7, 11]. We also re-examined the association of birth order and family size with obesity outcomes [21] but found no relation.

### Study outcomes

We used height and weight measured at military induction to define Body Mass Index (BMI: kg/m^2^) as a continuous measure and to define overweight and obesity for men with a BMI from 25 kg/m^2^ to <30 kg/m^2^ (‘overweight’) or 30 kg/m^2^ or more (‘obesity’), respectively. We determined which individuals had a weight/height ratio of 120% or more for comparison with previous analyses by Ravelli et al. [7].

### Statistical analysis

We estimated long-term famine effects using a Difference-in-Differences (DiD) design to capture interactions of place and time of birth. As noted, the place classification identifies three regions: the large West famine cities, the other cities in the West and rural West, and unexposed controls born outside the West. The birth months define exposures in early, mid, or late pregnancy trimesters. The control groups include men born in unexposed regions and unexposed months. We examined the relation of body size with famine exposure among individuals exposed in the early infancy and found no effects for this period. These individuals were merged with the other controls.

We examined differences in discrete outcomes (the odds of being overweight and/or obese) on a multiplicative scale in logistic regression models and on an additive scale using risk differences. We also examined differences in BMI as a continuous variable in linear regression models and used quantile regressions to examine the impact of famine exposure across the entire range of the BMI distribution.

We stratified by social class (sons of manual vs. non-manual workers) to control for potential confounding, in view of social class differences in BMI at age 19 and because the decline in births during the famine was especially pronounced among manual workers [13].

To analyze the study as a ‘randomized experiment’ to the extent possible, our first analyses were limited to sons of women who were already pregnant during the famine. Selecting only men born after July 1945, this approach excludes potential confounding by measured and unmeasured factors related to the likelihood of conceiving under famine conditions. As a second approach, we added men who were conceived during the famine as in a previous analysis of the study population [7]. We then compared outcomes from the two approaches.

## Results

### Demographics by region of birth

Table 1 shows selected characteristics for the study population of 371,100 men, classified as births in the West famine cities, births in the other cities or rural areas in the West, and births in the North, East, or South (Other Netherlands). The mean height of the conscripts was 177.4 cm and the mean BMI was 21.58 kg/m^2^. The proportion of men at age 19 with overweight is 6.3%, of men with a weight/height index over 120% is 1.6%, and of men with obesity is 0.4%.

**Table 1:** Selected characteristics for Dutch male conscripts born 1944–1947 and examined at age 19, by place of birth.

Birth place	Western Netherlands Famine cities	Western Netherlands other cities and rural	Other Netherlands cities and rural	All
Number of subjects (*n*)	94,249	73,913	202,938	371,100
Height (cm) (*SD*)	177.9 6.4	178.2 6.3	176.9 6.4	177.4 6.4
Body mass index (kg/m^2^) (*SD*)	21.6 2.3	21.6 2.2	21.6 2.2	21.6 2.2
Overweight (BMI ≥ 25.0 and < 30 kg/m^2^) (%)	6127 6.5	4381 5.9	12,692 6.3	23,200 6.3
Obesity (weight/height ≥ 120% of 1976 standard) (%)	1689 1.8	1045 1.4	3242 1.6	5976 1.6
Obese (BMI ≥ 30 kg/m^2^) (%)	441 0.5	250 0.3	859 0.4	1550 0.4
Father manual occupation (%)	45,323 48.1	35,477 48.0	106,456 52.5	187,256 50.5
First born (%)	35,219 37.4	22,003 29.8	60,335 29.7	117,557 31.7
Births in cities with 25,000 population or over in 1940 (%)	94,250 100	17,690 23.9	60,562 29.8	172,502 46.5

### Overweight and obesity by month and region of birth

Table 2 shows overweight, weight/height ratio exceeding 120% (‘Obese’ Ravelli 1976), and obesity status for the study population by selected birth months and region of birth. Being overweight was somewhat more common (6.5%) among recruits born in the Western Netherlands famine cities compared to recruits born in other parts of the West (5.9%) or elsewhere in the Netherlands (6.3%). In all regions, the proportion of individuals who were overweight or obese was higher in successive birth years.

**Table 2:** Overweight and obesity status by year and month and place of birth, Dutch male conscripts born 1944–1947 and examined at age 19.

	Western Netherlands Famine cities				Western Netherlands other cities and rural				Other Netherlands cities and rural				All
Birth year and month	Total births (*n*)	BMI ≥ 25 to < 30 (%)	‘Obese’ Ravelli 1976 (%)	BMI ≥ 30 (%)	Total births (*n*)	BMI ≥ 25 to < 30 (%)	‘Obese’ Ravelli 1976 (%)	BMI ≥ 30 (%)	Total births (*n*)	BMI ≥ 25 to < 30 (%)	‘Obese’ Ravelli 1976 (%)	BMI ≥ 30 (%)	Total births (*n*)
Jan–Oct 1944 Exposed to famine after birth	17,596	981 (5.6)	251 (1.4)	60 (0.3)	15,306	813 (5.3)	169 (1.1)	38 (0.2)	40,825	2,240 (5.5)	524 (1.3)	133 (0.3)	73,727
Nov 1944-Jan 1945 Trim 3 exposed	5023	255 (5.1)	50 (1.0)	11 (0.2)	4370	221 (5.1)	58 (1.3)	10 (0.2)	11,171	61717617 (5.5)	131 (1.2)	42 (0.4)	20,564
Feb–Apr 1945 Trim 2 and 3 exposed	5193	322 (6.2)	87 (1.7)	22 (0.4)	4884	290 (5.9)	58 (1.2)	10 (0.2)	13,265	898 (6.8)	195 (1.5)	39 (0.3)	23,342
May–Jul 1945 Trim 1, 2 and early 3rd exposed	4683	332 (6.9)	105 (2.2)	29 (0.6)	4487	250 (5.6)	52 (1.2)	15 (0.3)	12,159	735 (6.0)	173 (1.4)	45 (0.4)	21,329
Aug 1945–Jan 1946 Conceived during famine	5894	398 (6.8)	105 (1.8)	32 (0.5)	6309	370 (5.9)	87 (1.4)	22 (0.3)	24,124	1524 (6.3)	416 (1.7)	120 (0.5)	36,327
Feb–Dec 1946 Conceived and born after famine	31,430	2199 (7.0)	607 (1.9)	158 (0.5)	21,476	1366 (6.4)	350 (1.6)	91 (0.4)	54,392	3511 (6.5)	968 (1.8)	265 (0.5)	107,298
1947 Conceived and born after famine	24,430	1650 (6.8)	484 (2.0)	129 (0.5)	17,081	1071 (6.3)	271 (1.6)	64 (0.4)	47,002	3167 (6.7)	835 (1.8)	215 (0.5)	88,513
All births	94,249	6127 (6.5)	1689 (1.8)	441 (0.5)	73,913	4381 (5.9)	1045 (1.4)	250 (0.3)	202,938	12,692 (6.3)	3242 (1.6)	859 (0.4)	371,100

### Excess overweight by date and place of birth

In Table 3 (Upper Panel A) we present the relative odds for being overweight at age 19 from the Difference-in-Difference (DiD) place by time interaction terms for births in the Western famine cities (Famine) and births in the remainder of the West (West Other) relative to unexposed controls born in the North, East, or South.

**Table 3:** Odds Ratios (95% CI) for being Overweight (BMI ≥ 25 to < 30) after prenatal famine exposure starting in 1st, 2nd, or 3rd trimester of gestation. Dutch male conscripts born in 1944–1947 by father’s occupation. Comparing ‘Famine’ births in largest Western Netherlands cities and ‘West Other’ births to controls born outside Western Netherlands. (A) Births in gestation at the time of the famine. (B) All births 1944–1947.

Father’s Occupation		
Manual	Non-Manual	All*
*n* = 71,205	*n* = 67,757	*n* = 138,962
Trim 1+	Trim 1+	Trim 1+
Famine: 1.30 (1.06, 1.60)	Famine: 0.95 (0.75, 1.20)	Famine: 1.13 (0.97, 1.32)
West Other: 0.98 (0.78, 1.23)	West Other: 0.92 (0.72, 1.18)	West Other: 0.95 (0.80, 1.12)
Trim 2+	Trim 2+	Trim 2+
Famine: 0.90 (0.73, 1.11)	Famine: 0.90 (0.72, 1.12)	Famine: 0.90 (0.77, 1.04)
West Other: 0.95 (0.76, 1.18)	West Other: 0.84 (0.67, 1.07)	West Other: 0.90 (0.77, 1.06)
Trim 3	Trim 3	Trim 3
Famine: 0.79 (0.63, 1.00)	Famine: 1.05 (0.82, 1.35)	Famine: 0.90 (0.76, 1.06)
West Other: 0.92 (0.73, 1.16)	West Other: 0.97 (0.74, 1.28)	West Other: 0.94 (0.79, 1.12)

Father’s Occupation		
Manual	Non-Manual	All*
*n* = 187,254	*n* = 183,846	*n* = 371,100
Trim 1+	Trim 1+	Trim 1+
Famine: 1.25 (1.04, 1.50)	Famine: 0.91 (0.73, 1.13)	Famine: 1.09 (0.95, 1.25)
West Other: 1.01 (0.82, 1.24)	West Other: 0.90 (0.72, 1.13)	West Other: 0.96 (0.82, 1.12)
Trim 2+	Trim 2+	Trim 2+
Famine: 0.86 (0.71, 1.04)	Famine: 0.86 (0.71, 1.05)	Famine: 0.87 (0.75, 0.99)
West Other: 0.98 (0.81, 1.19)	West Other: 0.83 (0.67, 1.03)	West Other: 0.91 (0.79, 1.05)
Trim 3	Trim 3	Trim 3
Famine: 0.76 (0.62, 0.94)	Famine: 1.01 (0.80, 1.26)	Famine: 0.86 (0.74, 1.01)
West Other: 0.95 (0.76, 1.18)	West Other: 0.96 (0.75, 1.23)	West Other: 0.95 (0.81, 1.12)

Famine births: ten Western Netherlands famine cities; West other: remaining births in Western Netherlands. Control population: births outside Western Netherlands. No births excluded from the study. For classification details see Appendix1.

*Adjusted for occupation status.

These odds for being overweight reflect interactions on a multiplicative scale. The odds were significantly elevated for sons of manual workers born in the Western cities after famine exposure starting in the first trimester of gestation (OR = 1.30; 95% CI: 1.06–1.60) and lower after exposure in the third trimester (OR = 0.79; 95% CI: 0.63–1.00). There were no deviations from unity for other study groups. Adding births conceived during the famine, the OR for being overweight after the first-trimester gestation in this group was 1.25 (95% CI: 1.04–1.50), and 0.76 (95% CI: 0.62–0.94) for being overweight after exposure in the third trimester. (Table 3, Lower Panel B). Our study findings did not change when the Ravelli study population [7] comparing ‘Famine area’ births to ‘Control area’ births was used in the analysis (Supplementary Table 2). The findings of analyses performed in Table 3 were the same (to the second decimal point) if obese men were added to the overweight group.

In view of the possible non-linearity of the interaction effect in logistic models [22] and to evaluate possible interactions on additive scales, we also calculated the Relative Excess Risk due to Interaction (RERI) and the Attributable Proportion (AP) of the risk in the doubly exposed group (i.e. exposed both by place of birth and date of birth) [23], In Supplementary Tables 3a and 3b we show the risk of being overweight at age 19 in births in gestation at the time of the famine and in all births 1944–1947. Both RERI and AP were in close agreement with interaction terms obtained from logistic regression analysis.

For obesity defined by a BMI of 30 kg/m^2^ or more, the relative odds for individuals exposed in the first trimester of gestation in Western famine cities (sons of manual workers) was 1.29 (95% CI: 0.70–2.38) among all births, based on small numbers (0.4% prevalence). For obesity defined as a height to weight ratio of 120% or more as in the Ravelli study, the odds for obesity were also significantly elevated among sons of non-manual workers born in Western famine cities (OR 1.58; 95% CI: 1.01–2.47).

### Continuous and quantile regression outcomes

After stratification for social class and region, there were no height differences between men exposed to famine in the different pregnancy trimesters and controls.

The mean BMI increase for individuals exposed to famine starting in the first trimester of gestation is 0.23 kg/m^2^ (95% CI: 0.15–0.31). The increase is consistent across all quantiles but larger in the upper tail of the distribution (Supplementary Fig. 2). There was no mean BMI increase after the second-trimester exposure (difference 0.06 kg/m^2^; 95% CI: −0.02 to 0.14) and no difference across quantiles (Supplementary Fig. 3). There also was no mean BMI increase after the third-trimester exposure (mean difference 0.04 kg/m^2^; 95% CI: −0.04 to 0.12), but an increase was seen in the lower tail of the BMI distribution and significant decreases in the extreme upper tail of the BMI distribution (Supplementary Fig. 4).

### Comparison with other exposure definitions

We evaluated the robustness of our famine exposure definition by comparison with previous classifications based on distributed food rations during the famine [10, 11] or as used in the 1976 obesity study [7] (Supplementary Fig. 1). All definitions point towards elevated odds for being overweight or obese after famine exposure in early gestation for births in the Western famine cities: Cohort T2 as defined by food rations; cohort D1 as defined in the 1976 obesity study; and cohort Trim 1+ as defined in the current study (Supplementary Tables 4a, 4b, and 4c).

### Sensitivity analysis

We examined the robustness of the above study findings to limiting analyses to the subset of all city births and to using alternative controls defined as births either in the North/East or South or both regions combined. We compared findings using either pre-famine or post-famine births as controls. The findings did not change significantly using any of these approaches.

## Discussion

We set out to examine differences in body size in young men after prenatal and infant famine exposure. There were no differences in height as noted before in this study population [7] and also for a smaller non-military population sample in the 1990s [24]. We expressed weight differences as BMI differences as the preferred indicator of overweight and obesity [25] and of excess body fat associated with long-term morbidity and mortality. Conscripts were examined in the early 1960s when the prevalence of overweight in young men in the Netherlands was low (6.3%).

We found an increase in being overweight (OR = 1.30, 95% CI: 1.06–1.60) after prenatal famine exposure early in gestation (Table 3, Upper Panel A). The increase was limited to sons of manual workers born in famine cities of the Western Netherlands. The impact of BMI increases in young adulthood on later mortality is considerable: in a subset of 45,000 recruits in this cohort, being overweight at age 19 was associated with a 30% increase in overall mortality through age 63 relative to those with a BMI between 19–25 kg/m^2^ [11]. Worldwide, a consistent association is seen between being overweight BMI or higher and increased all-cause mortality [26].

Contrary to the weight/height based obesity findings reported by Ravelli et al. [7], we found no increase in overweight defined by BMI in sons of non-manual workers exposed to early gestation in the famine cities (Table 3A: OR 0.95, 95% CI: 0.75–1.20). When we apply the Ravelli obesity measure to our study population we do however find a difference (OR 1.58; 95% CI 1.01–2.47). As the Ravelli obesity measure [7] corresponds to a BMI between 27–28 kg/m^2^, this shows that different measures of body adiposity can lead to different estimates of the relation between prenatal famine and adiposity at age 19.

In our analysis, the estimated increase in overweight in sons of manual workers is only marginally lower after the addition of births who had been conceived during the famine (OR = 1.25, 95% CI: 1.04–1.50) (Table 3, Lower Panel B). This shows that in this population there was little need to consider ‘selective fertility’ as even large differences in fertility by the social class during the famine did not substantially change the estimate of long-term overweight risk [27].

Our comparisons show no long-term effects on being overweight in sons of non-manual workers born in the famine. This confirms at age 19 that even inside the famine area there existed substantial differences in access to food by social class as earlier noted by contemporary accounts [27]. Stratification by social class (manual vs. non-manual) is needed however to quantify the differences in long-term outcomes.

BMI associations with prenatal famine starting in the first trimester of pregnancy were consistent across the entire BMI spectrum but even more pronounced at the extreme upper tail of the BMI distribution (Supplementary Fig. 2). BMI associations with prenatal famine in the third trimester of pregnancy were not consistent. From a statistical perspective, there was no association overall, although quantile regressions show an upward shift in the lower tail of the BMI distribution and a downward shift in the upper tail (Supplementary Fig. 4). Reported findings of a reduced risk of overweight and obesity therefore only reflect a downward BMI shift in the upper tail. The literature on the long-term health impact of overweight differences in this population is limited to one report, showing increased mortality up to age 63 years among men with a BMI of 25 kg/m^2^ or more, but similar mortality in men with a BMI of <19, 19–20, or 20–25 kg/m^2^ [11]. From a biological perspective, therefore, only an increase but not a decrease in overweight and obesity has been associated with later health changes. Our findings demonstrate again that differences across the entire BMI distribution should be taken into account when interpreting associations between early famine and dichotomized measures defined by BMI at higher cut-off points.

There was no increase in BMI or overweight for births in the West outside the large famine cities. This confirms that the burden of the famine was concentrated in the large cities in the West as was previously suggested by contemporary accounts [14] but not yet confirmed in national data.

Our findings did not change when limiting controls to births born before the famine (pre-famine controls) or after the famine (post-famine controls). This reflects that in the Dutch famine there were no long-term BMI differences among births exposed to famine in the first year of life.

We compared our findings using alternative exposure classifications based on either weekly food rations of [10, 11] or the classification of the 1976 obesity study [7]. All exposure classifications point towards the beginning of gestation as the critical exposure period (Supplementary Tables 4a, 4b, and 4c). Our study findings are also robust to the use of different exposure definitions for prenatal famine and robust to the choice of different unexposed regions selected as controls.

Causal explanatory pathways remain speculative. Some data show that the impact of prenatal famine on BMI at age 58 years can be mediated by changes in DNA methylation [28]. Differences in DNA methylation also suggest that fetal deaths may be increased by prenatal famine exposure [29]. With regard to other outcomes, studies of coronary artery disease in men and women followed through middle age after prenatal exposure to the Dutch famine present conflicting findings [30, 31]. The men in the current study exposed in early gestation show a 10% increase in mortality; however, at age 63 no increase in deaths reported from cardiovascular disease [10, 11].

Among the many strengths of our study we mention (a) standardized measurements of height and weight during military examinations at age 19 of entire male birth cohorts in the Netherlands, and (b) classification of famine exposure by place and date of birth for all men. This study also has some limitations. As an example, our BMI measure is widely used in population studies but does not differentiate between lean and fat tissue.

The military examinations at age 19 did not include waist or hip circumference or other measures of body mass distribution, which might be better indicators of chronic disease risk. In our clinical study of middle-aged individuals born in three famine exposed clinics in the Netherlands, these measures were highly correlated. However, further studies by others are needed to determine the performance of alternatives to a BMI-based index of body mass distribution in predicting chronic disease risk.

In summary, our findings demonstrate that the long-term adverse impact of prenatal famine on later life Type 2 diabetes and mortality is already presenting in young adults as a significant increase in overweight risk.

## Supplementary information

Supplementary materials
