# Supplementary material for: Overweight and obesity at age 19 after pre-natal famine exposure
Source: Int J Obes (Lond). 2021 May 10;45(8):1668–76. doi: 10.1038/s41366-021-00824-3 (PMC8310790; doi:10.1038/s41366-021-00824-3)
Supplement: Supplementary file 1 — Supplementary materials [file 41366_2021_824_MOESM1_ESM.docx]

**Supplement Table 1**

**Prenatal exposure to the Dutch famine**

**Alternative classifications based on month of birth**

| **Classification criterion** | **Authors’ designation of prenatal exposure period** | **Month of Birth** | **Outcome studied** | **Reference** |
| --- | --- | --- | --- | --- |
| **Famine exposure during specific trimesters of pregnancy defined by stage of pregnancy in relation to Nov 1, 1944** | Trim 3: 3^rd^ trimester  Trim 2+: 2^nd^ and 3^rd^ trimester  Trim 1+: 1^st^, 2^nd^ and early 3^rd^ trimester | Nov 1944 – Jan 1945  Feb 1945 – Apr 1945  May 1945 – Jul 1945 | - Overweight (BMI>=25)  - Obese (Ravelli 1976)  - Obesity (BMI>=30) | This study |
| **Famine exposure during specific trimesters of pregnancy defined by average of food rations not exceeding 900 kcal/day in that trimester** | T3: 3^rd^ trimester  T2: 2^nd^ trimester  T1: 1^st^ trimester | Feb 1945 – Jun 1945  May 1945 – Sep 1945  Aug 1945 – Dec 1945 | - Survival age 18-63 years  - Cause of death | Ekamper 2014,  Ekamper 2015 |
| **Individuals grouped by month of birth to test for the presence of critical periods of famine exposure “with adequate numbers”** | B1: 3^rd^ trimester  B2: 2^nd^/3^rd^ trimester  D1: 1^st^/ 2nd trimester  D2: 1^st^ trimester | Nov 1944 – Jan 1945  Feb 1945 – May 1945  Jun 1945 – Sep 1945  Oct 1945 – Jan 1946 | - Obese (Ravelli 1976) | Ravelli 1976 |

**Supplement Table 2**

**Odds Ratios (95% CI) for being Overweight (BMI ≥25 to <30)**

**after prenatal famine exposure starting in 1^st^, 2^nd^, or 3^rd^ trimester of gestation**

**Dutch male conscripts born in 1944-1947**

**by father’s occupation**

**Comparing ‘Famine area’ births to ‘Control area’ births, excluding births in non-urbanized rural areas (Ravelli 1976)**

**A) Births in gestation at the time of the famine**

| **Father’s Occupation** | |  |
| --- | --- | --- |
| **Manual** | **Non-Manual** | **All*** |
| **n=55,645** | **N=48,076** | **n=103,721** |
| Trim 1+  Famine: 1.30 (1.05, 1.61). | Trim 1+  Famine: 1.06 (0.83, 1.36) | Trim 1+  Famine: 1.19 (1.01, 1.40) |
| Trim 2+  Famine: 0.83 (0.67, 1.04) | Trim 2+  Famine: 0.99 (0.79, 1.25) | Trim 2+  Famine: 0.90 (0.77, 1.06) |
| Trim 3  Famine: 0.80 (0.63, 1.02) | Trim 3  Famine: 1.13 (0.87, 1.47) | Trim 3  Famine: 0.93 (0.78, 1.11) |

**B) All births 1944-1947**

| **Father’s Occupation** | |  |
| --- | --- | --- |
| **Manual** | **Non-Manual** | **All*** |
| **n=148,128** | **n=135,878** | **n=284,006** |
| Trim 1+  Famine: 1.25 (1.03, 1.51) | Trim 1+  Famine: 0.99 (0.79, 1.24) | Trim 1+  Famine: 1.13 (0.98, 1.31) |
| Trim 2+  Famine: 0.80 (0.66, 0.98) | Trim 2+  Famine: 0.92 (0.75, 1.13) | Trim 2+  Famine: 0.86 (0.74, 0.99) |
| Trim 3  Famine: 0.77 (0.62, 0.96) | Trim 3  Famine: 1.05 (0.83, 1.34) | Trim 3  Famine: 0.88 (0.75, 1.04) |

Famine area: Seven Western Netherlands famine cities; Control area: Remaining births in urbanized and urbanized rural areas in Western or non-Western Netherlands. Births in non-urbanized rural areas nationwide excluded from study. For details see Appendix 1.

*Adjusted for Occupation status.

**Supplement Table 3a**

**Risk of being overweight (BMI ≥25 to <30) at age 19**

**after prenatal famine exposure from the first trimester of gestation,**

**Sons of manual workers, by region of birth**

**A) Births in gestation at the time of the famine**

|  |  | **Overweight Incidence**  **by Trim 1+ Exposure and Region** | |  |
| --- | --- | --- | --- | --- |
| **Famine cities**  **births** | **Trim 1 date of birth** | **Overweight**  **(n)** | **Population**  **at risk (n)** | **Overweight (%)** |
| West Famine cities | Yes | 191 | 2,310 | 8.27 |
| West Other cities and rural | Yes | 132 | 2,148 | 6.15 |
| Non West cities and rural | Yes | 406 | 6,544 | 6.20 |
| West Famine cities | No | 837 | 13,915 | 6.02 |
| West Other cities and rural | No | 720 | 11,913 | 6.04 |
| Non West cities and rural | No | 2,107 | 34,375 | 6.13 |
| All  births |  | 4,393 | 71,205 | 6.17 |

**Measures of interaction on the additive scale**

| Comparison | Measure | Point estimate | 95% CI | p-value |
| --- | --- | --- | --- | --- |
| West Famine cities  vs Non West cities and rural | RERI^1^  AP^2^ | 0.39  0.28 | 0.15 to 0.62  0.14 to 0.42 | 0.0016  0.0001 |
| West Other cities and rural  vs Non West cities and rural | RERI^1^  AP^2^ | -0.06  -0.06 | -0.29 to 0.16  -0.29 to 0.17 | 0.5804  0.5940 |

^1^ RERI: Relative Excess Risk due to Interaction.

^2^ AP: Attributable Proportion: proportion of the risk in the doubly exposed group (Famine city births exposed from 1^st^ trimester) that is due to interaction.

Measures as defined by VanderWeele and Knol (2014) using entries from the above Table.

**Supplement Table 3b**

**Risk of being overweight (BMI ≥25 to <30) at age 19**

**after prenatal famine exposure from the 1^st^ trimester of gestation,**

**Sons of manual workers, by region of birth**

**B) All births 1944-1947**

|  |  | **Overweight Incidence**  **by Trim 1+ Exposure and Region** | |  |
| --- | --- | --- | --- | --- |
| **Famine cities**  **births** | **Trim 1 date of birth** | **Overweight**  **(n)** | **Population**  **at risk (n)** | **Overweight (%)** |
| West Famine cities | Yes | 191 | 2,310 | 8.27 |
| West Other cities and rural | Yes | 132 | 2,148 | 6.15 |
| Non West cities and rural | Yes | 406 | 6,544 | 6.20 |
| West Famine cities | No | 3,083 | 43,012 | 7.17 |
| West Other cities and rural | No | 2,201 | 33,329 | 6.60 |
| Non West cities and rural | No | 6,749 | 99,911 | 6.76 |
| All  births |  | 12,762 | 187,254 | 6.82 |

**Measures of interaction on the additive scale**

| Comparison | Measure | Point estimate | 95% CI | p-value |
| --- | --- | --- | --- | --- |
| West Famine cities  vs Non West cities and rural | RERI^1^  AP^2^ | 0.27  0.21 | 0.06 to 0.48  0.07 to 0.36 | 0.0134  0.0028 |
| West Other cities and rural  vs Non West cities and rural | RERI^1^  AP^2^ | 0.01  0.02 | -0.18 to 0.20  -0.19 to 0.22 | 0.8793  0.8779 |

^1^ RERI: Relative Excess Risk due to Interaction.

^2^ AP: Attributable Proportion: proportion of the risk in the doubly exposed group (Famine city births exposed from 1^st^ trimester) that is due to interaction.

Measures as defined by VanderWeele and Knol (2014) using entries from the above Table.

**Supplement Table 4a**

**Odds Ratios (95% CI) for being Overweight (BMI ≥25 to <30) after prenatal famine exposure**

**Dutch male conscripts born in 1944-1947, by month and region of birth**

**(All births; n=371,100)**

| **Month of Birth** | **Famine exposure classification** | | | |
| --- | --- | --- | --- | --- |
|  |  |  | |  |
|  | **This study** | **Ekamper 2014, 2015** | | **Ravelli 1976** |
|  |  |  | |  |
| 1944/11 | Trim 3  Famine: 0.86 (0.74, 1.01)  West Other: 0.95 (0.81, 1.12) |  |  | Cohort B1  Famine: 0.87 (0.74, 1.01)  West Other: 0.95 (0.80, 1.11) |
| 12 |  |  |  |  |
| 1945/1 |  |  |  |  |
| 2 | Trim 2+  Famine: 0.87 (0.75, 0.99)  West Other: 0.91 (0.79, 1.05) | T3  Famine: 0.90 (0.80, 1.01)  West Other: 0.91 (0.80, 1.03) |  | Cohort B2  Famine: 0.93 (0.82, 1.04)  West Other: 0.91 (0.80, 1.03) |
| 3 |  |  |  |  |
| 4 |  |  |  |  |
| 5 | Trim 1+  Famine: 1.09 (0.95, 1.25)  West Other: 0.96 (0.82, 1.12) |  | T2  Famine: 1.14 (1.00, 1.31)  West Other: 0.96 (0.83, 1.10) |  |
| 6 |  |  |  | Cohort D1  Famine: 1.09 (0.95, 1.24) West Other: 0.91 (0.79, 1.05) |
| 7 |  |  |  |  |
| 8 |  | T1  Famine: 1.01 (0.87, 1.17)  West Other: 0.97 (0.83, 1.13) |  |  |
| 9 |  |  |  |  |
| 10 |  |  |  | Cohort D2  Famine: 1.01 (0.84, 1.23)  West Other: 1.05 (0.87, 1.26) |
| 11 |  |  |  |  |
| 12 |  |  |  |  |
| 1946/1 |  |  |  |  |

Famine: Western Netherlands famine cities; West Other: Western Netherlands other cities and rural.

*Unexposed controls: Non-Western Netherlands cities and rural births 1944-1947.

**Supplement Table 4b**

**Odds Ratios (95% CI) for ‘Obese’ (Ravelli 1976) prevalence after prenatal famine exposure**

**Dutch male conscripts born in 1944-1947, by month and region of birth**

**(All births; n=371,100)**

| **Month of Birth** | **Famine exposure classification** | | | |
| --- | --- | --- | --- | --- |
|  |  |  | |  |
|  | **This study** | **Ekamper 2014, 2015** | | **Ravelli 1976** |
|  |  |  | |  |
| 1944/11 | Trim 3  Famine: 0.77 (0.55, 1.07)  West Other: 1.29 (0.93, 1.77) |  |  | Cohort B1  Famine: 0.77 (0.55, 1.08)  West Other: 1.28 (0.93, 1.77) |
| 12 |  |  |  |  |
| 1945/1 |  |  |  |  |
| 2 | Trim 2+  Famine: 1.03 (0.79, 1.34)  West Other: 0.91 (0.67, 1.24) | T3  Famine: 1.02 (0.81, 1.28)  West Other: 0.93 (0.72, 1.21) |  | Cohort B2  Famine: 1.06 (0.84, 1.33)  West Other: 0.96 (0.73, 1.24) |
| 3 |  |  |  |  |
| 4 |  |  |  |  |
| 5 | Trim 1+  Famine: 1.43 (1.11, 1.85)  West Other: 0.92 (0.67, 1.27) |  | T2  Famine: 1.40 (1.09, 1.79)  West Other: 0.95 (0.71, 1.27) |  |
| 6 |  |  |  | Cohort D1  Famine: 1.41 (1.12, 1.78) West Other: 0.86 (0.64, 1.15) |
| 7 |  |  |  |  |
| 8 |  | T1  Famine: 0.89 (0.67, 1.17) West Other: 0.95 (0.70, 1.28) |  |  |
| 9 |  |  |  |  |
| 10 |  |  |  | Cohort D2  Famine: 0.88 (0.61, 1.27)  West Other: 0.95 (0.66, 1.38) |
| 11 |  |  |  |  |
| 12 |  |  |  |  |
| 1946/1 |  |  |  |  |

Famine: Western Netherlands famine cities; West Other: Western Netherlands other cities and rural.

*Unexposed controls: Non-Western Netherlands cities and rural births 1944-1947.

**Supplement Table 4c**

**Odds Ratios (95% CI) for Obesity (BMI>=30) prevalence after prenatal famine exposure**

**Dutch male conscripts born in 1944-1947, by month and region of birth**

**(All births; n=371,100)**

| **Month of Birth** | **Famine exposure classification** | | | |
| --- | --- | --- | --- | --- |
|  |  |  | |  |
|  | **This study** | **Ekamper 2014, 2015** | | **Ravelli 1976** |
|  |  |  | |  |
| 1944/11 | Trim 3  Famine: 0.54 (0.27, 1.06)  West Other: 0.76 (0.37, 1.52) |  |  | Cohort B1  Famine: 0.54 (0.28, 1.06)  West Other: 0.73 (0.36, 1.49) |
| 12 |  |  |  |  |
| 1945/1 |  |  |  |  |
| 2 | Trim 2+  Famine: 1.33 (0.78, 2.28)  West Other: 0.86 (0.42, 1.76) | T3  Famine: 1.23 (0.79, 1.91)  West Other: 0.94 (0.54, 1.63) |  | Cohort B2  Famine: 1.29 (0.82, 2.02)  West Other: 0.77 (0.42, 1.42) |
| 3 |  |  |  |  |
| 4 |  |  |  |  |
| 5 | Trim 1+  Famine: 1.55 (0.95, 2.51)  West Other: 1.14 (0.61, 2.04) |  | T2  Famine: 1.39 (0.88, 2.21)  West Other: 1.00 (0.55, 1.81) |  |
| 6 |  |  |  | Cohort D1  Famine: 1.53 (0.99, 2.35) West Other: 0.91 (0.51, 1.61) |
| 7 |  |  |  |  |
| 8 |  | T1  Famine: 0.96 (0.57, 1.60)  West Other: 0.72 (0.38, 1.37) |  |  |
| 9 |  |  |  |  |
| 10 |  |  |  | Cohort D2  Famine: 0.88 (0.44, 1.76)  West Other: 0.89 (0.43, 1.84) |
| 11 |  |  |  |  |
| 12 |  |  |  |  |
| 1946/1 |  |  |  |  |

Famine: Western Netherlands famine cities; West Other: Western Netherlands other cities and rural.

*Unexposed controls: Non-Western Netherlands cities and rural births

**Supplement Figure 1**

**Prenatal exposure to the Dutch famine**

**Alternative classifications based on month of birth**


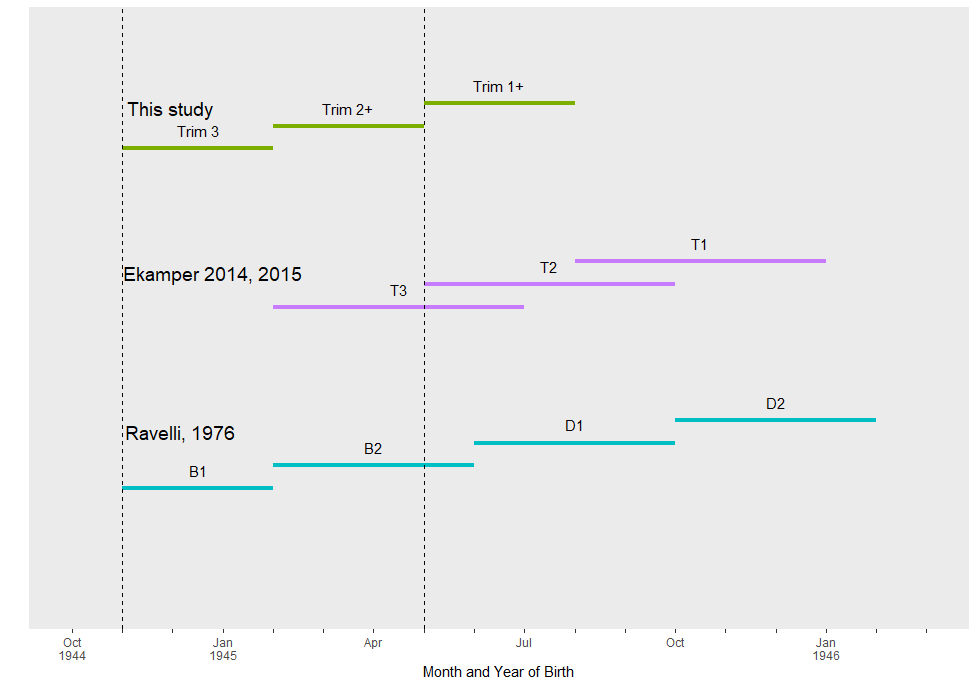


**Supplement Figure 2**

**BMI changes among Trim 1 exposed cohorts relative to unexposed controls**

**by BMI quantile, point estimates and 95% CI**


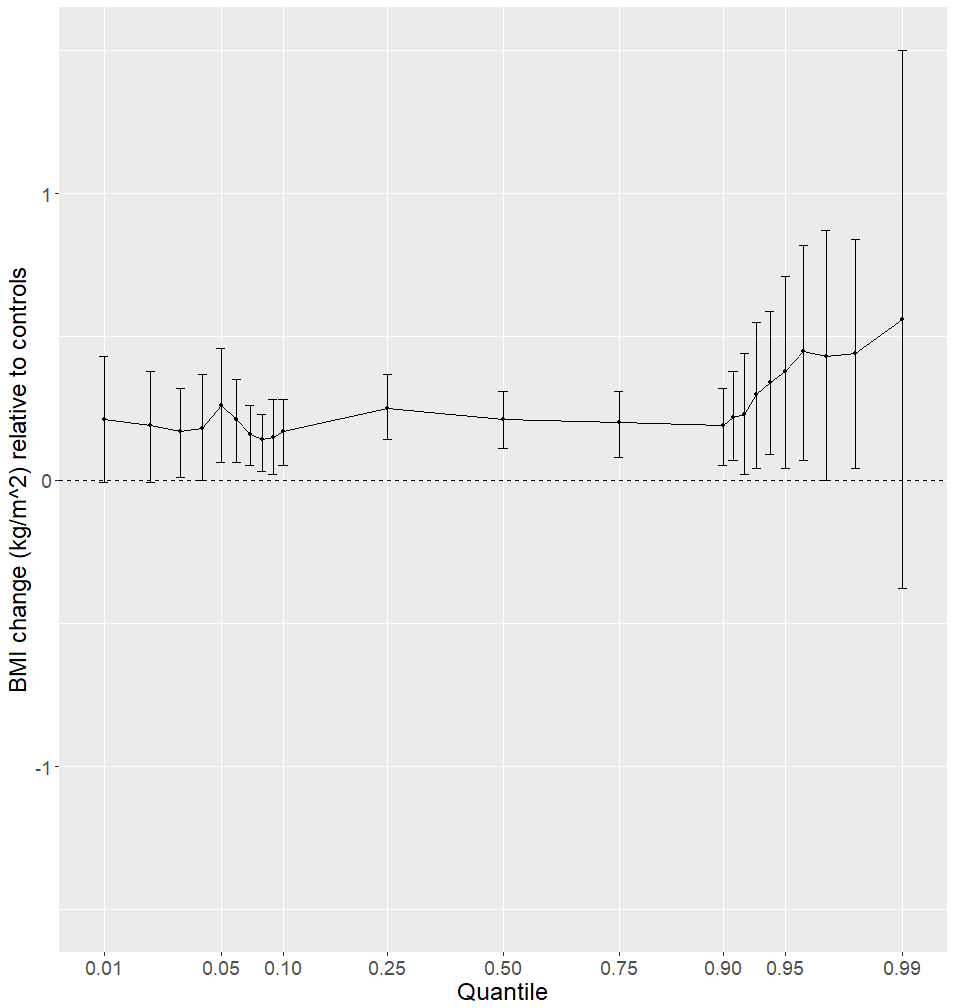


**Supplement Figure 3**

**BMI changes among Trim 2 exposed cohorts relative to unexposed controls**

**by BMI quantile, point estimates and 95% CI**


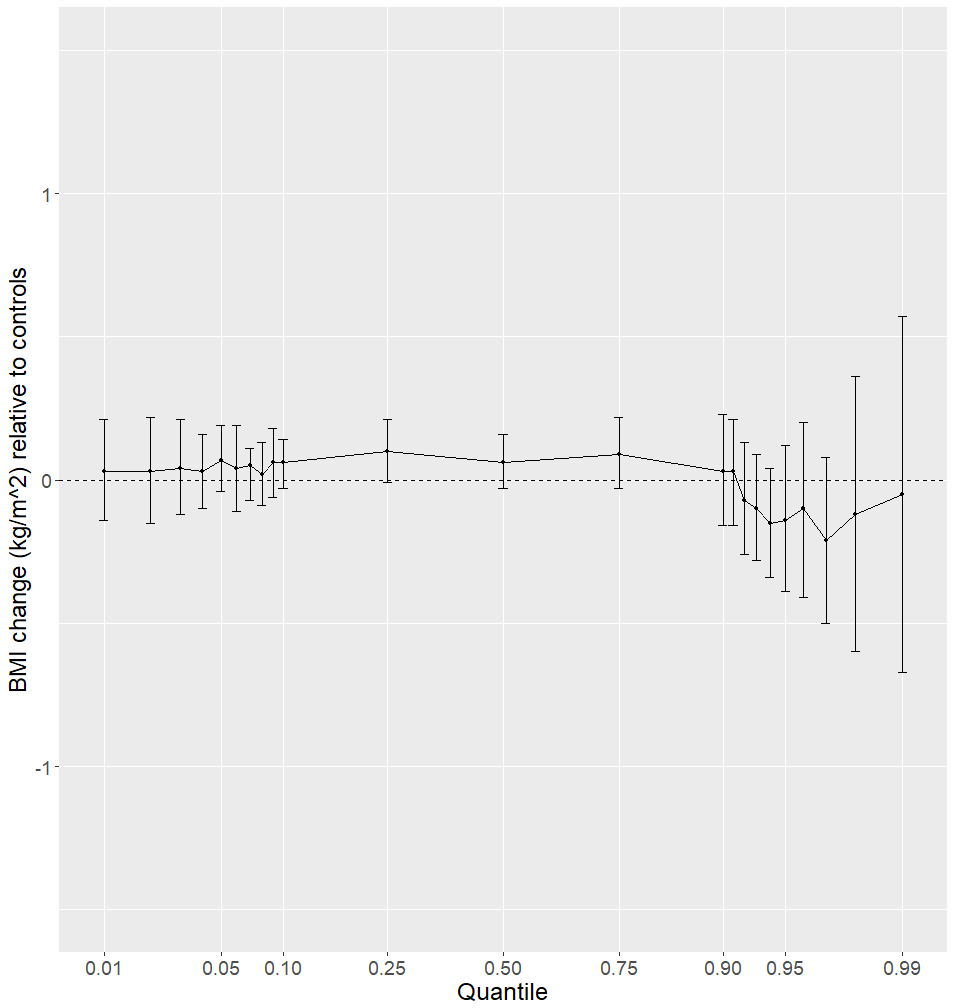


**Supplement Figure 4**

**BMI changes among Trim 3 exposed cohorts relative to unexposed controls**

**by BMI quantile, point estimates and 95% CI**


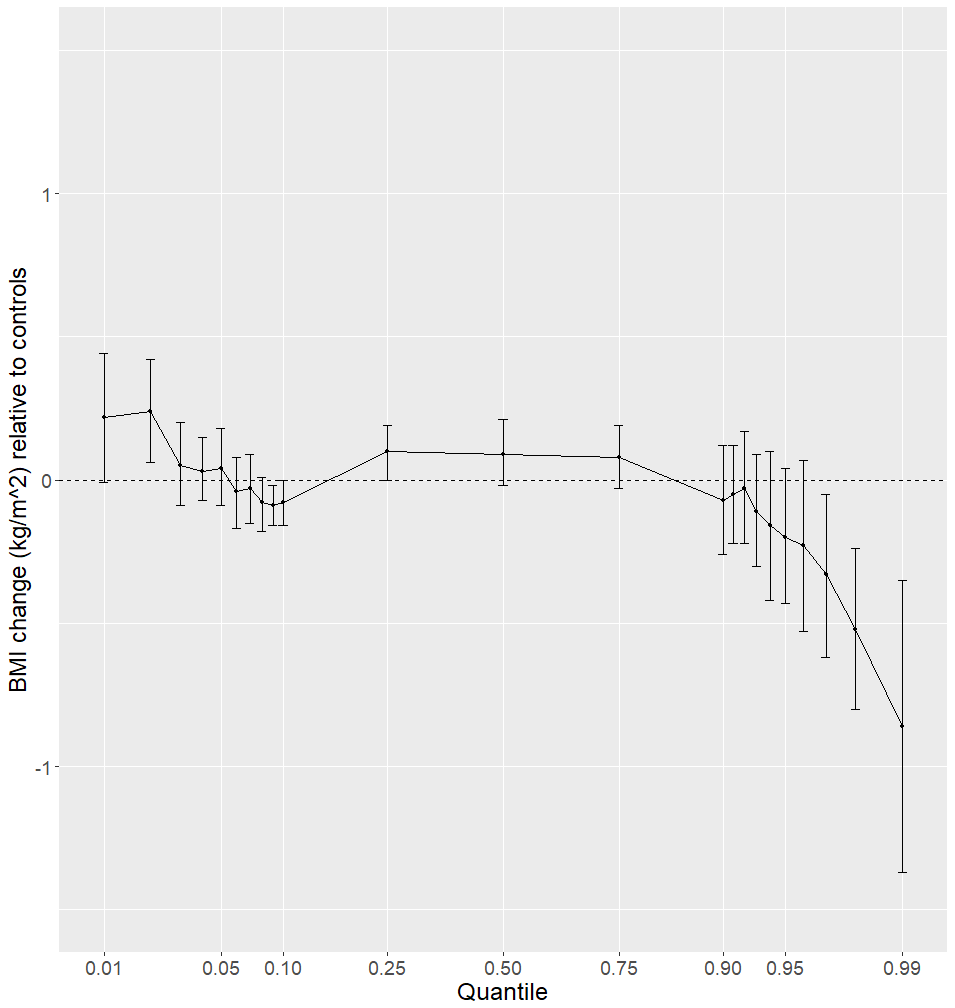


**Appendix 1**

**Grouping by Place of Birth**

We classified all men as births in three regions. We distinguished

1. births in the West famine cities: Amsterdam, Delft, Den Haag, Haarlem, Leiden, Rotterdam, Schiedam, Utrecht, Vlaardingen and Voorburg;

2. births in the other Western cities (Alkmaar, Amersfoort, Bussum, Dordrecht, Gouda, Den Helder, Hilversum, Velsen, Zaandam, Zeist) and rural areas;

3. births in the North/South/Eastern cities (Almelo, Apeldoorn, Arnhem, Bergen op Zoom, Breda, Deventer, Eindhoven, Enschede, Groningen, Helmond, Hengelo, ‘s-Hertogenbosch, Kerkrade, Leeuwarden, Maastricht, Nijmegen, Roosendaal, Tilburg, Venlo, Zwolle) and North/South/Eastern rural areas combined.

Ravelli (1976) classied the men in two groups:

1. Famine area births: in the West famine cities Amsterdam, Delft, Den Haag, Haarlem, Leiden, Rotterdam, and Utrecht.

2. Control area births: in any area in the Netherlands defined as urban or urbanized rural in 1947 by the Dutch census typology of municipalities by urbanization level. Non-urbanized rural births were not included for study. (*Centraal Bureau voor de Statistiek, 1983. Typologie van de Nederlandse gemeenten naar urbanisatiegraad op 28 februari 1971. Hoofdafdeling sociale rekeningen, Staatsuitgeverij, ‘s-Gravenhage)*

**Appendix 2**

**Grouping by Month of Birth**

Using distributed food rations, we quantified famine exposure during specific trimesters of pregnancy based on famine rations not exceeding 900 kcal/day on average in this trimester. (Ekamper et al., 2015) This provides 5-month time windows. By this definition, individuals exposed in the first trimester of gestation were born in Aug-Dec 1945 (T1); those exposed in the second trimester in May-Sept 1945 (T2); and those exposed in the third trimester in Feb-Jun 1945 (T3). These cohorts span five months, and adjacent cohorts have two months of overlap as individuals will meet the famine definition for two trimesters of pregnancy.

In a prior obesity study (Ravelli et al., 1976), the recruits were grouped in cohorts by month of birth “according to the period of exposure during gestation and early postnatal life, to test for the presence of critical periods with adequate numbers” as follows: exposed in the first trimester of gestation: births Oct-Dec 1945 (D2); exposed in the first and second trimester: births Jun-Sept 1945 (D1); exposed in the second and third trimester: births Feb-May 1945 (B2); exposed in the third trimester: births Oct 1944-Jan 1945 (B1). In this classification, the birth cohorts span four months, except for cohort D2 that spans three months.
